# Methodology insights and population characteristics of the Nutri-Bébé 2022 study: study design, sample description, and data collection

**DOI:** 10.3389/fnut.2026.1784566

**Published:** 2026-03-23

**Authors:** Jean-Pierre Chouraqui, Sandra Brancato, Florent Vieux, Floriane Poulain

**Affiliations:** 1 Paediatric Division of Nutrition and Gastro-enterology, Pediatric Department, Grenoble-Alpes University Hospital (CHUGA), Grenoble, France; 2 Association Française de Pédiatrie Ambulatoire, Brignon, France; 3 MS-Nutrition, UMR C2VN (Inserm, Inrae, AMU), Faculté de Médecine La Timone, Marseille, France; 4 Secteur Français des Aliments de l’Enfant (SFAE), Alliance7, Paris, France

**Keywords:** dietary data collection, infants, nutrition survey methodology, observational study, representative sampling, socio-economic status, young children

## Abstract

**Background/objectives:**

Regular assessment of dietary patterns and nutrient intake during early life is essential for establishing appropriate recommendations to promote healthy eating behaviors in children. The objective of the Nutri-Bébé 2022 study was to update these data in a nationally representative sample of infants and young children (0–3 years) living in mainland France, and to investigate potential associations between socio-economic factors and feeding practices. This article describes the study methodology and the population sample.

**Methods:**

This observational cross-sectional study selected a random sample using the quota sampling method. After recruitment and informed consent, parents participated in an initial face-to-face interview. In the following days they completed an online food consumption questionnaire over three non-consecutive days, including a weekend day, followed by a questionnaire on eating behavior. To ensure representativeness the sample was calibrated and weighted. The total sample comprised 1,224 children divided into 11 age groups: an initial sample of 880 parent–child dyads plus an oversampling of some child subgroups to assess their nutritional intakes.

**Results:**

Detailed characteristics of parents, households and children, including anthropometric data and socio-economic status are described. Among mothers, 5% were under 25 years of age and 39.7% were over 34 years of age, 85.6% were living as a couple and 52.1% had a non-tertiary level of education. Of the included children, 50.1% were boys, 59.4% had siblings and 160 were breastfed. Breastfeeding prevalence was 35.2% up to 3 months, 13.7% at 6 months and 10.4% at one year. In the total sample, 947 children consumed complementary foods, with a mean age of introduction of 5.46 ± 2.04 months.

**Conclusion:**

The data obtained from a representative sample using a valid methodology should allow for future detailed analysis of dietary patterns and macro- and micronutrient intake.

**Clinical trial registration:**

ClinicalTrials.gov, NCT05649527.

## 1 Introduction

Early childhood, which includes infants (up to and including 11 months) and young children, also called toddlers (from 12 up to and including 35 months of age), is a vulnerable period marked by more changes in growth, development, eating patterns, nutritional needs, gut functions, and microbiome development than any other time of life (1, 2). Early feeding practices influence the development of several aspects of children’s eating behavior, including the acquisition of food preferences, self-regulatory skills, and future health (3–7). The latest results from the ongoing Global Burden of Disease Study show that six of the top 11 risk factors driving the global burden of non-communicable diseases (NCDs) are related to diet (8). Early life nutrition is particularly associated with the risk of developing chronic NCDs, which constitute a major public health challenge and are the leading cause of disease, disability, and mortality worldwide (9–13).

The periodic nationwide assessment of dietary patterns and nutrient intake provides essential data to inform policy decisions aimed at improving nutrition and health and for developing guidelines (14, 15). These nutrition surveys use standard epidemiological methods and aim to obtain a representative study population (16). However, this can be challenging (17). Through its European Food and Nutrition Action Plan, the World Health Organization (WHO) called on countries to conduct national nutrition surveys, emphasizing the importance of obtaining reliable, representative, and standardized data (18). In 2018, 88% of European countries reported conducting such surveys, but few reported data on infants and young children (9).

Since 1981, and every eight years thereafter (1981, 1989, 1997, 2005, and 2013), the “Secteur Français des Aliments de l’Enfance” (SFAE) has organized and funded a representative national survey of infants’ and young children’s nutrition (19–23). These surveys assessed feeding practices and nutrient intake in infants and young children in France. Over the years, the sample size has increased to include children up to three years of age and those who were breastfed (23). In addition, the methodology has evolved to strengthen the study in light of existing recommendations on food surveys and to account for changes in lifestyles and the evolution of commercial products. To update the data collected in the Nutri-Bébé 2013 survey (23), the sixth survey was conducted in 2022 (Nutri-Bébé 2022), following the latest European Food Safety Authority (EFSA) guidelines (24). The recruitment period was delayed by one year due to the Covid-19 pandemic, which could have affected the availability of parents and stakeholders as well as children’s dietary patterns (25–28). The Nutri-Bébé 2022 survey was conducted to assess the food consumption patterns, feeding practices, and nutrient intake of French infants and young children, and to analyze the socio-demographic factors that may be associated with these data.

This article aims to (i) present and discuss the methodological aspects of the Nutri-Bébé 2022 survey, from the development of the study design and sampling plan to data collection, and (ii) describe the included population sample. The manuscript also discusses the challenges of conducting a study of this scope and complexity and compares it to similar international studies.

## 2 Methods

### 2.1 General organization of the study

#### 2.1.1 Study design and implementation

As in previous Nutri-Bébé studies, the 2022 survey is a national cross-sectional observational survey conducted between March 10 and July 20, 2022, in mainland France (i.e., excluding Corsica, overseas departments, regions, communities, and New Caledonia). It was conducted by the official polling institute IPSOS under the supervision of a panel of experts.

#### 2.1.2 Expert panel composition

The expert group included two pediatric gastroenterologists and nutritionists, six food scientists, one nutrition scientist, one sociologist, one dietitian, and several statistical experts. They participated at different stages of the study and were responsible for the protocol, designing the questionnaires, and analyzing the data.

#### 2.1.3 Compliance and methodological guidelines

The study was designed and conducted in compliance with the updated EFSA guidelines of the EU Menu methodology (24). The report is based on recommendations from the statement on Strengthening the Reporting of Observational Studies in Epidemiology – Nutritional Epidemiology (STROBE-nut) (29).

#### 2.1.4 Organizations in charge of data collection and analysis

IPSOS collected and analyzed the consumption data. CREDOC (Centre de Recherche pour l’Étude et l’Observation des Conditions de Vie; Research Center for the Study and Observation of Living Conditions), a nonprofit government organization, calculated, reported, and analyzed nutrient intakes based on feed intakes. MS-Nutrition, a research and consulting company hosted by the laboratory C2VN “Center of Research in Cardiovascular and Nutrition” (INRA/INSERM/AMU) within the Timone Faculty of Medicine in Marseille, completed the analyses and statistics. All steps were completed under the supervision of the Expert Panel.

### 2.2 Recruitment and sampling

#### 2.2.1 Recruitment approach

##### 2.2.1.1 Investigator engagement with parents

Trained IPSOS investigators randomly approached parents of children under three years old as they were leaving a day care center, walking in the street, or visiting a public park. This was conducted in various regions of mainland France, in urban areas of different sizes, in different districts of the same city, and in rural areas.

##### 2.2.1.2 Quota sampling method

The quota sampling method was gradually applied to select the population sample. This method was based on population characteristics from the 2015 census of the National Institute for Statistics and Economic Research (INSEE, Institut National de la Statistique et des Études Économiques) (30). The applied quota considered the following parameters: residential area, agglomeration size, mother’s activity status (active or inactive before any maternity leave), referent’s occupational and socio-occupational status (“Professions et Catégories Socio-professionnelles,” PCS, classified into three categories: PCS+, PCS−, and inactive as detailed in Table 1), number of children in the household, and the children’s age and gender. According to INSEE, the reference person in the family is the oldest employed person in the household (31).

**Table 1:** Detailed description of the socio-occupational categories (PCS).

Categories	Occupations
PCS +	Farmers Craftsmen Shopkeepers and similar positions Liberal professionals Entrepreneurs and corporate executives Intermediate corporate administrative or commercial positions Engineers, foremen, supervisors Senior public service managers Intermediate teaching, healthcare, public service positions and similar professions Intellectual or artistic professions
PCS –	Farm workers Skilled workers Specialized workers Commercial employees Corporate administrative employees Public service employees Personal services employees
Inactive	Student Unemployed Without a profession Retired

#### 2.2.2 Eligibility criteria

##### 2.2.2.1 Parent eligibility

To be eligible, responding parents (hereafter referred to as “parent”) had to (i) be over 18 years old, (ii) have an eligible child, (iii) be primarily responsible for the child’s meals, (iv) have sufficient French-language proficiency, (v) have access to the Internet, and (vi) know how to use a computer.

##### 2.2.2.2 Child eligibility

Eligible children had to (i) be born full-term (37–42 weeks gestational age) with a weight above 2.5 kg, (ii) be aged 0.5 to 35 months at the time of the study, (iii) be non-institutionalized, (iv) be free of any intercurrent or chronic disease (recorded in the child’s health book or reported by parents), and (v) take meals on the days of food data collection at home or in a day care center. If two eligible children under 3 years old were present in the same household, only one was selected within the quota.

#### 2.2.3 Sample stratification and oversampling

##### 2.2.3.1 Age group division

The objective of this first stage was to recruit 880 children divided into eleven age groups to align with previous surveys and the key stages of child development: 0.5–3, 4, 5, 6, 7, 8–9, 10–11, 12–17, 18–23, 24–29, and 30–35 months. Each age bracket was defined by the first day of the starting month and the last day of the ending month. The age group was determined based on the child’s age at the first interview.

##### 2.2.3.2 Oversampling for specific subgroups

In addition to this recruitment, specific oversampling was conducted using the same criteria to ensure a sample of 80 non-breastfed children in each age group, as well as 60 consumers of Young Child Formula (YCF) and 60 non-consumers in each age group over 1 year old (referred to as the ‘total sample’ hereafter). The purpose of this oversampling was to analyze in detail their nutritional intakes, which were compared to those of non-consumers of YCF.

### 2.3 Weighting

The national representativeness of the final samples was ensured by two successive weightings. The first weighting was applied to the initial sample, using the recruitment quota as weighting factors. To improve the representativeness of the samples, the following additional weighting factors were included: PCS, parental level of education, and cross-weighting between age and sex (so that for each age group, there were equal numbers of girls and boys). The age weighting aimed to rebalance the size of each age group to ensure it was as representative as possible of the French population. The second weighting was applied to the total sample (i.e., after oversampling non-breastfed children and YCF consumers and non-consumers) and further accounted for the prevalence of breastfed children and YCF consumers based on those reported in the initial sample after the first weighting.

### 2.4 Conduct of the study and data collection

#### 2.4.1 Initial contact and face-to-face interview

During the first contact, the investigator explained the purpose and design of the survey, invited the parent to participate, and, after obtaining agreement, collected their contact details (telephone number and email address) and scheduled a face-to-face interview at home. The investigator provided the parent with the link to the survey information letter (https://www.fw.ipsos.com/ftp/NUTRIBEBE/Informations_parents.pdf). A few days later, the investigator followed up with the parent to confirm the appointment. During the meeting, the investigator verified that the parent and child met the eligibility criteria and that the parent had read the information note and obtained agreement from the second holder of parental authority to participate in the study. An initial questionnaire listing the parent’s and child’s characteristics was then administered using the CAPI® software (Computer Assisted Personal Interviews; https://www.idsurvey.com/en/capi-survey-software/).

#### 2.4.2 Anthropometric measurements

The child’s weight and length or height were recorded from the child’s health book if the data were sufficiently recent (i.e., less than 15 days earlier for infants 0.5–5 months, less than 1 month earlier for infants 6–11 months, or less than 2 months earlier for children 12 months and older) and consistent with WHO standards (32). If the weight and/or height were either outdated or clearly inconsistent with the child’s age according to WHO standards (i.e., greater than mean +3 SD or less than mean −3 SD), the child was weighed (naked, with a clean diaper or underwear) with the parent or alone if able to stand, using a Be You By-ebs01 scale with a precision of 100 g. The child was measured lying down before 24 months of age or standing if older, using a measuring tape and a square with a precision of 0.5 cm. If parents did not allow the investigator to perform these measurements, the child was not included in the study. Weights and BMI values that remained clearly inconsistent with the child’s age after measurement (i.e., greater than mean +3 SD or less than mean −3 SD), possibly due to measurement or reporting errors, were considered “unreliable” and were excluded from the calculation of mean weight and from nutritional analyses expressed per kilogram of body weight, as in the previous study (23).

#### 2.4.3 Food diary and consumption questionnaire

At the end of the interview, the investigator and the parent selected three days (two weekdays and one weekend day) within the next two weeks to complete the online consumption questionnaire. He varied the days for each household to distribute them evenly across the weekdays and a weekend day. He gave the parent a paper model consumption diary to record all foods and drinks given during the day, and a photographic toolkit to assess food portions (although using a scale was recommended whenever possible to determine the exact quantities consumed by the child). A printed copy of the questionnaire was provided to the day care center staff or the nanny if necessary, so they could report the child’s consumption, which the parents would then enter online. The photographic toolkit was the same as the one validated in the 2013 study (23). Afterwards, the interviewer sent an invitation email to the respondent (the parent who identified as primarily responsible for preparing meals) to confirm inclusion in the study and provided the link to access the online consumption questionnaire, a video tutorial, and a list of Q&As, which were regularly updated as parents provided feedback.

#### 2.4.4 Final online questionnaire and follow-up

After the three days of food diary were completed and validated, a link was sent to the parent to access the final phase of the survey: an online questionnaire focused mainly on the child’s feeding background, physical activity, screen exposure, and purchasing preferences. If necessary, the investigator made several telephone reminders between each phase. Respondents could refuse to answer certain questions or discontinue participation in the study at any time. In these cases, the parent–child dyad was excluded from the study and replaced by another participant with the same characteristics for the defined quota. Exclusion criteria also included: (i) loss to follow-up during the study, (ii) non-compliance with the protocol, and (iii) taking too long to complete the various questionnaires (more than one month between the face-to-face interview and the completion of the food diary). Parents who completed all three steps received a voucher worth €20.

### 2.5 Collection of population characteristics

During the face-to-face interview and in the online questionnaire, information on household and children’s characteristics was collected and directly entered by the investigator using the CAPI software.

#### 2.5.1 Household characteristics

These included the number of people and children in the household, marital status, age and gender of the respondent, occupation (PCS), and level of education of the respondent as well as those of the reference person in charge of the child if different, economic status, and source of advice regarding the child’s diet and lifestyle. The employment status of the respondent and the reference person was classified according to the INSEE definition (31) as ‘working’ or ‘inactive’. The level of education included three categories: (i) no education or primary and/or early secondary school; (ii) up to the baccalaureate (high school diploma); and (iii) higher education including university (tertiary education). The economic status of the household was assessed using both net household income and the parents’ financial perception (Table 2).

**Table 2:** Parents’ and household characteristics.

Characteristics	Reference population INSEE 2015 (30)	Initial sample before/after weighting *n* = 880		Final sample before/after weighting *n* = 1,224	Weighted non-breastfed sample *n* = 1,064
Area of recruitement					
Paris region	22.0%	18.9/22.0%		17.9/21.4%	20.2%
Northwest	22.0%	22.2/22.0%		23.8/22.3%	22.9%
Northeast	22.0%	26.5/22.0%		23.0/22.2%	22.0%
Southwest	10.0%	8.7/10.0%		10.7/10.1%	10.6%
Southeast	24.0%	23.7/24.0%		24.7/24.0%	24.3%
Urban size					
Paris agglomeration	78.0%	18.4/21.1%	78.0%	17.3/19.8%	18.8%
≥ 100,000 inhabitants		45.2/42.8%		48.6/44.0%	44.2%
[20,000–99,999] inhabitants.		4.8/4.1%		4.2/4.7%	4.9%
< 20,000 inhabitants		10.7/10.0%		10.8/9.5%	10.5%
Rural	21.0%	20.9/22.0%		19.0/21.9%	21.6%
Mother’s age					
< 25 years	8.0%	7.0/4.7%		7.4/5.0%	5.0%
25–29 years	26.0%	25.9/20.1%		25.8/19.4%	18.3%
30–34 years	34.0%	33.5/33.7%		33.4/32.8%	32.1%
≥ 35 years	33.0%	31.1/39.1%		30.6/39.7%	41.1%
Unknown		2.4/2.4%		2.8/3.1%	3.4%
Respondent’s age ^a^					
< 25 years	8.0%	7.2/4.7%		7.5/5.0%	5.0%
25–29 years	26.0%	26.4/20.4%		26.4/19.8%	18.8%
30–34 years	34.0%	34.0/34.1%		34.1/33.5%	32.8%
≥ 35 years	33.0%	32.5/40.8%		32.0/41.8%	43.4%
Family status					
Married	90.0%	44.4/47.7%	85.9/88.5%	43.1/45.6%	46.2%
Partnership		41.5/40.8%		40.8/39.8%	39.2%
Separated or divorced	10.0%	14.1/11.5%		4.3/5.0%	5.1%
Widowed				0.3/0.2%	0.3%
Single				11.5/9.3%	9.1%
Mother’s parental leave					
Full time		30.3/32.5%		29.3/30.5%	29.7%
Part-time		7.2/7.5%		6.5/6.2%	6.5%
No		36.2/36.2%		36.5/39.6%	40.7%
No answer/Unknown		26.2/23.8%		27.6/23.6%	23.1%
Respondent parental leave ^a^					
Full time		30.2/32.5%		29.1/30.3%	29.5%
Part-time		7.0/7.2%		6.4/6.0%	6.2%
No		37.3/37.4%		37.6/40.9%	42.2%
No answer		25.4/23.0%		27.0/22.8%	22.2%
Mother’s education level					
Primary or early secondary school	15.0%	2.2/1.8%		2.4/3.2%	3.1%
Up to the baccalauréat	38.0%	36.1/37.1/%		37.1/49.0%	49.6%
Upper education	47.0%	61.1/60.6%		60.1/47.7%	47.1%
No answer		0.6/0.4%		0.4/0.2%	0.1%
Respondent’s educational level ^a^					
Primary or early secondary school		2.2/1.8%		2.4/3.2%	3.1%
Up to the baccalauréat		36.1/36.9%		37.2/48.9%	49.5%
Upper education		61.4/61.1%		60.1/47.7%	47.2%
No answer		0.3/0.2%		0.2/0.1%	0.2%
Mother’s socio-occupational status					
PCS +	39.0%	27.1/27.2%		27.5/37.2%	38.1%
PCS –	38.0%	45.0/47.2%		42.7/36.9%	36.3%
Inactive	23.0%	25.6/23.2%		27.0/22.8%	22.2%
Unknown		2.3/2.4%		2.7/3.0%	3.4%
Respondent’s socio-occupational status ^a^					
PCS +		28.2/28.5%		28.6/38.8%	39.8%
PCS –		46.2/48.3%		44.3/38.4%	38.0%
Inactive		25.6/23.2%		27.1/22.9%	22.2%
Referent’s socio-occupational status^b^					
PCS +	47.0%	44.4/46.4%		43.4/46.9%	48.3%
PCS –	47.0%	43.4/47.6%		44.7/46.7%	46.1%
Inactive	6.0%	12.2/6.0%		11.9/6.3%	5.6%
Net household income					
< 1700 €		14.1/11.2%		14.7/12.5%	12.0%
[1700–1999] €		6.1/6.0%		6.4/5.6%	6.1%
[2000–2,299] €		7.9/8.5%		7.7/9.0%	9.5%
[2300–2,999] €		18.4/17.5%		17.2/15.8%	15.4%
[3000–4,499] €		25.2/29.6%		24.7/28.4%	28.4%
[4500–5,399] €		6.7/7.4%		6.5/7.0%	6.9%
≥ 5,400 €		3.2/3.4%		3.7/4.7%	4.6%
Refusal to respond		18.3/16.3%		19.1/16.9%	17.0%
Financial perception by the parents					
Comfortable		15.1/15.5%		14.0/14.8%	14.9%
Going well		39.0/38.7%		39.1/38.0%	37.3%
Struggling		18.2/19.4%		18.8/20.0%	20.2%
Must be careful		19.1/18.9%		19.0/19.3%	19.4%
Difficult to achieve		7.6/6.7%		7.7/6.2%	6.4%
Indebted or needing help		0.4/0.3%		0.7/0.8%	0.9%
Refusal to respond		0.6/0.5%		0.6/0.8%	0.9%

^a^: The mother was the respondent in 97.2% of the cases. ^b^: The referent was the oldest active person in the household.

#### 2.5.2 Children’s characteristics

The children’s characteristics included date of birth, birth weight, child feeding (i.e., people responsible for feeding or preparing meals, time spent and frequency of meal preparation, frequency of milk bottle consumption, method of food preparation and feeding, past or current breastfeeding), as well as the child’s physical activity and screen time.

### 2.6 Dietary information collection method

Dietary information was collected as closely as possible to the method recommended by EFSA (24). Parents completed their child’s consumption diary online over three non-consecutive days (two weekdays and one weekend day). Parents were asked to complete the diary online on the days planned by the investigator during the face-to-face interview. If the child was sick on one of the planned days, the parent was advised to choose another day, ensuring the distribution rule was respected. The report was a structured questionnaire that documented all feeding practices and provided detailed information on the consumption of foods, beverages, and vitamin supplementation.

#### 2.6.1 Development and use of the digital food diary

The food diary was developed with a panel of experts using a digital tool produced by id+Technology (https://www.idplus-technology.com/) and based on the questionnaire used in 2013 (23). The digital tool was specifically developed for the needs of the study and is not publicly accessible. All consumption (breast milk/formula, food, water, and other liquids) had to be recorded by the parent using the tool. For each entry, information was required on the food consumed and the context of consumption: time of consumption; occasion (breakfast, morning, lunch, afternoon, dinner, after dinner/before bedtime, night); name of the food or drink (selected from a list with intuitive entry, or entered in a free field if not found in the list); brand or origin (for unbranded foods); detailed recipe for homemade preparations; total quantity offered to the child; and quantity remaining after the child had eaten or drunk. For exclusively breastfed children (children fed exclusively breast milk, without any additional food or drink until the introduction of complementary foods, and afterward, those whose only milk source was their mother’s milk), as well as for partially breastfeed children (children fed both breast milk and formula), the data were limited to time and frequency because it was not possible to assess the volume and composition of the mother’s milk consumed.

#### 2.6.2 Quality control and follow-up procedures

The tool was designed to automatically remind parents of forgotten items, such as drinking water and added salt. If there were doubts regarding the record, the investigator contacted the parent in the following days. If the doubt persisted, the child was excluded from the sample.

### 2.7 Statistical analyses

Results were expressed as mean ± standard deviation (SD). When relevant, median, first and third quartiles (Q1, Q3), as well as minimum and maximum values, were reported in the text within brackets or in tables. Statistical analyses were performed using SAS Statistical Software version 9.4 (SAS Institute Inc., Cary, NC, USA). Categorical variables were compared using the Chi-Square (Χ^2^) test. Student T-tests were used to compare quantitative characteristics (e.g., body weight) between groups. When relevant, individual weighting factors were applied to ensure the representativeness of the final samples. The threshold for statistical significance was set at *p* < 0.05.

## 3 Results

### 3.1 Course of the survey

#### 3.1.1 Target population and recruitment

According to the INSEE 2015 census, 1,948,351 mothers were in the target population defined by the inclusion criteria of the Nutri-Bébé 2022 survey (30). Among the offspring of this eligible population, 1,342 (0.7‰) children were recruited. Less than one fourth of this recruitment occurred at the end of winter, more than 50% during spring, and less than 25% at the beginning of summer.

#### 3.1.2 Final sample

**Figure 1:**
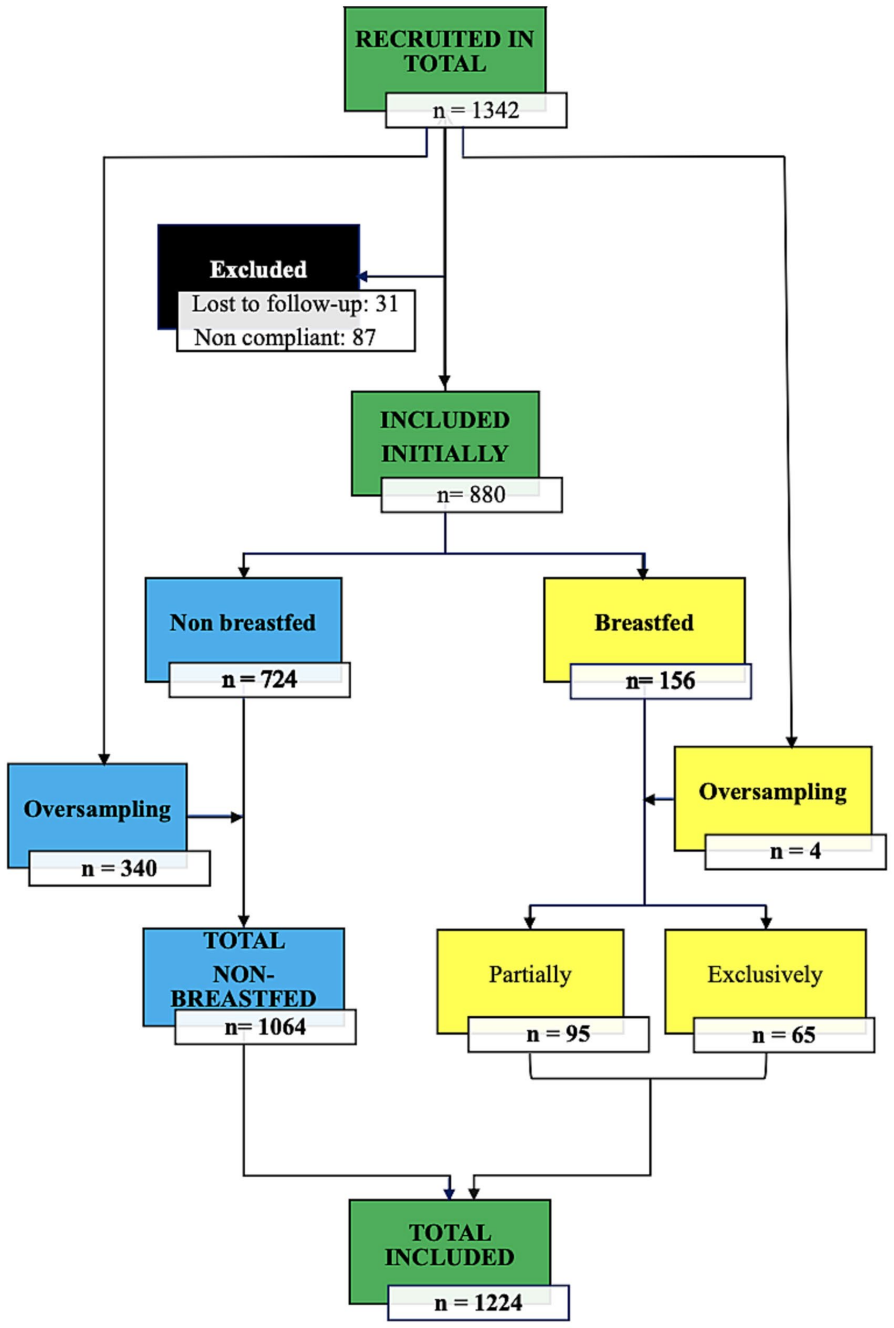
Recruitment and inclusion flowchart.

A total of 1,224 (91.2%) children were included in the study due to full compliance with the inclusion and non-inclusion criteria, including obtaining informed consent from both parents (Figure 1). This final sample included 880 children initially recruited plus 344 recruited as an oversample. The reasons for excluding 118 children were loss to follow-up for 31 and non-compliance with the protocol for 87.

#### 3.1.3 Respondent

The mother was the respondent in most cases (97.2%), while the father was the respondent in 2.8% of cases.

#### 3.1.4 Conduct of the study and time spent

The initial face-to-face interview at the respondent’s home took an average of 70 min. The respondent spent an average of 29 min completing the online questionnaire. On average, 10 telephone reminders were necessary for each respondent to clarify and specify certain points.

### 3.2 Parents’ and household characteristics

The characteristics of the included parents and households are presented in Table 2, along with those of the reference population (30). This demonstrates the increased representativeness achieved through successive weighting. The mean age of the responding mothers was 31.98 ± 5.45 years (*n* = 1,190; median 32; Q1–Q3 = 28–36; range 18–49), and 56.64% were primiparous. The responding fathers (*N* = 34) had a mean age of 34.26 ± 6.81 years (median 33; Q1–Q3 = 29–39; range 21–39). The mean age of the 1,032 responding non-breastfeeding women at the time of inclusion was 32.10 ± 5.49 years (median 32; Q1–Q3 = 28–36; range 19–49), whereas that of breastfeeding mothers (n = 158) was 31.18 ± 5.10 years (median 31; Q1–Q3 = 28–35; range 18–43), *p* = 0.035.

### 3.3 Children characteristics

#### 3.3.1 Sample overview

The characteristics of the included children are presented in Table 3. Comparison with the reference population confirms improved representativeness (30). The sample included 707 infants and 517 young children. There were 609 boys and 615 girls (sex ratio: 0.99). More than 40% were the only child in the household.

**Table 3:** Children characteristics.

Characteristics	Reference population INSEE 2015 (30)	Initial sample before/after weighting *n* = 880	Final sample before/after weighting *n* = 1,224	Non-breastfed final sample before/after weighting *n* = 1,064
Age groups (months)				
[0.5–3]	10.0%	9.1/10.0%	10.2/10.0%	7.6/5.8%
[3–4]	3.0%	9.1/3.0%	8.8/3.2%	7.4/2.5%
[4–5]	3.0%	9.1/3.0%	7.6/3.1%	7.3/3.0%
[5–6]	3.0%	9.1/3.0%	8.3/3.2%	8.3/3.1%
[6–7]	3.0%	9.1/3.0%	7.3/3.1%	7.1/2.8%
[7–9]	5.0%	9.1/5.0%	7.8/5.0%	8.1/5.1%
[9–11]	5.0%	9.1/5.0%	7.7/5.0%	8.1/5.2%
[11–17]	18.0%	9.1/18.0%	12.7/17.7%	13.2/17.8%
[17–23]	16.0%	9.1/16.0%	10.4/15.9%	11.4/17.2%
[23–29]	17.0%	9.1/17.0%	9.8/16.8%	10.7/17.9%
[29–35]	17.0%	9.1/17.0%	9.4/16.9%	10.8/19.5%
Gender				
Boys	50.0%	50.0/50.0%	49.7/50.1%	49.6/50.1%
Girls	50.0%	50.0/50.0%	50.2/49.9%	50.4/49.8%
Number of Children at home				
1	40.0%	55.6/40.0%	56.6/40.6%	56.7/40.7%
>1	60.0%	44.4/60.0%	43.4/59.4%	43.3/59.3%
Children in alternating custody		9.5/7.6%	9.2/7.9%	8.5/6.7%

#### 3.3.2 Birth and feeding status

All children were born at term, and the mean birth weight for the total sample was 3.293 ± 0.410 kg (*n* = 1,207; median: 3.250; interquartile range: 3.000–3.570; range: 2.500–4.800). Before over-sampling (*n* = 880), 18.2% of the children were breastfed at the time of inclusion, and 36.8% had been breastfed before the study, resulting in a total prevalence of 55% for currently or previously breastfed children. In the total sample (*n* = 1,224), the prevalence of breastfeeding by age group was 35.2% in the 0.5–3 months group, 26.8% at 4 months, 16.1% at 5 months, 13.7% at 6 months, 14.6% at 7 months, 10.4% at 8–9 months, 8.5% at 10–11 months, 9.7% at 12–17 months, 4.7% at 18–23 months, 5% at 24–29 months, and 0.0% at 30–35 months. Considering children who were breastfed before the study and those breastfed at the time of the study, the mean duration of exclusive breastfeeding (*n* = 290) was 167 ± 160 days (median: 101; Q1–Q3: 61–177; range: 1–915). Of these children, 243 subsequently consumed formula and 47 had partial breastfeeding (mean duration: 99 ± 99 days; median 30; Q1–Q3 = 30–184; range 4–402). Partial breastfeeding initiated at birth (*n* = 122) lasted an average of 100 ± 93 days (median 69; Q1–Q3 = 30–114; range 1–396).

#### 3.3.3 Distribution of the children’s weight

Of all children included, 1,064 were not breastfed at the time of the investigation (Figure 1) and will be the focus of a later report on nutrient intake. The age distribution of breastfed (*n* = 160) and non-breastfed children (*n* = 1,064) is shown in Table 4, with no difference between genders. The sex ratio was 1.

**Table 4:** Age (in days) distribution in breastfed (BF, *n* = 160) and non-breastfed (NBF, *n* = 1,064) children in each age group, by gender.

Age group (months)	Feeding	Gender	n	Mean	SD	Minimum	Q1	Median	Q3	Maximum
[0.5–3]	BF	Boy	25	60.7	27.3	16	36	65	78	117
		Girl	19	65.2	31.7	17	28	61	92	111
	NBF	Boy	37	74.0	29.4	19	53	71	98	119
		Girl	44	62.7	28.0	14	36	64	76	120
[3–4]	BF	Boy	12	132.4	7	122	124	132	137	144
		Girl	17	139	10.1	121	126	143	147	148
	NBF	Boy	40	131.0	8.3	121	123	128	136	149
		Girl	38	135.7	9.8	121	125	136	143	150
[4–5]	BF	Boy	8	158.9	5	151	155	157	158	168
		Girl	7	157.8	8.6	151	151	151	164	173
	NBF	Boy	33	163.7	8.7	152	155	161	171	180
		Girl	45	161.5	8.6	151	154	158	167	180
[5–6]	BF	Boy	7	196.1	10.6	181	185	192	206	208
		Girl	7	196.1	10.1	183	186	192	201	211
	NBF	Boy	42	194.9	8.0	181	188	194	201	211
		Girl	46	191.9	8.8	181	184	189	198	211
[6–7]	BF	Boy	9	226.1	8.8	212	219	224	236	237
		Girl	4	221.9	8.1	212	212	217	225	233
	NBF	Boy	31	223.9	7.3	213	216	224	227	239
		Girl	45	223.5	8.0	212	216	221	229	240
[7–9]	BF	Boy	5	276.6	10.9	262	265	271	277	299
		Girl	5	261.2	22.9	244	244	247	256	299
	NBF	Boy	49	268.3	16.7	242	251	271	277	301
		Girl	37	266.2	18.4	243	248	263	284	302
[9–11]	BF	Boy	3	331.5	13.8	316	316	323	336	348
		Girl	5	322.5	7.7	303	320	323	325	331
	NBF	Boy	38	326.6	15.8	303	311	322	338	359
		Girl	48	323.8	16.0	303	310	320	335	362
[11–17]	BF	Boy	6	476	35.1	381	468	481	485	524
		Girl	9	457	44.9	368	423	467	485	504
	NBF	Boy	79	436.5	50.9	365	380	438	481	542
		Girl	61	438.2	55.1	364	382	424	481	540
[17–23]	BF	Boy	3	672.6	68.6	585	585	651	704	740
		Girl	3	609.8	56.6	549	549	572	596	724
	NBF	Boy	65	619.8	57.3	546	568	612	652	738
		Girl	56	640.2	55.8	557	593	630	690	737
[23–29]	BF	Boy	3	902.7	23.6	826	854	896	909	916
		Girl	3	797.8	41.7	748	748	776	815	844
	NBF	Boy	58	821.7	52.5	743	768	825	860	922
		Girl	56	833.2	54.5	743	789	821	883	922
[29–35]	BF	Boy	0							
		Girl	0							
	NBF	Boy	55	1015.2	50.8	925	967	1,029	1,056	1,106
		Girl	60	1017.2	45.5	928	986	1,021	1,055	1,098

#### 3.3.4 Anthropometric measurements

**Figure 2:**
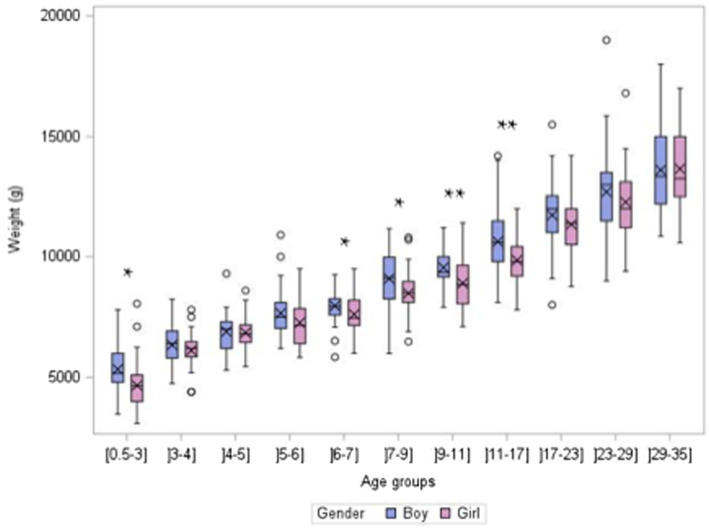
Weight distribution according to gender within age group in non-breastfed children (*n* = 1,050, after exclusion of unreliable weights).

Most weights (95.6%) and lengths/heights (91.5%) were obtained from the health book. For 14 children (7 boys and 7 girls), the weight was considered unreliable as defined above and is not included in Figure 2. The weight distribution of non-breastfed boys and girls is compared in Figure 2. In the (0.5–3 months) group and among those aged 6 to 17 months, boys were heavier than girls, while no difference in mean age was found. Of the total children included (*n* = 1,224), 4.66% had a body weight below the 3rd percentile of the WHO curves, and 3.76% had a weight above the 97th percentile according to age (32). Length/height and BMI by age group are presented in Table 5.

**Table 5:** Length/height (cm) and BMI (Kg/m^2^) distributions in non-breastfed children after exclusion of unreliable weights (*n* = 1,050).

Age (Months)	*n*	Height/BMI	Mean	SD	Minimum	Q1	Median	Q3	Maximum
[0.5–3]	81	Length	56.70	4.40	48.00	52.92	56.33	58.51	70.00
		BMI	15.35	2.39	8.91	14.02	15.13	16.68	22.26
[3–4]	77	Length	62.60	4.12	55.00	59.78	62.56	63.94	79.00
		BMI	15.95	1.86	11.22	14.47	16.02	17.19	20.48
[4–5]	77	Length	64.25	2.91	58.00	61.69	63.99	65.88	71.00
		BMI	16.66	1.52	13.38	15.50	16.66	17.65	20.11
[5–6]	87	Length	66.16	3.28	53.00	63.29	65.50	68.03	75.00
		BMI	17.09	1.91	13.10	15.83	17.04	17.80	28.48
[6–7]	76	Length	67.60	3.39	62.00	64.58	67.27	68.92	86.00
		BMI	17.03	1.87	8.45	16.06	17.13	17.64	22.17
[7–9]	85	Length	70.67	3.49	61.00	68.00	70.24	71.88	83.00
		BMI	17.69	1.99	12.27	16.35	17.73	18.30	24.13
[9–11]	86	Height	73.33	4.01	65.00	70.26	72.41	74.41	86.00
		BMI	17.22	1.93	12.32	15.78	17.20	18.29	21.32
[11–17]	137	Length	78.10	4.59	68.00	74.79	77.26	79.97	93.00
		BMI	16.93	2.31	11.32	15.12	17.07	18.17	25.95
[17–23]	121	Length	84.33	4.71	70.00	81.02	83.94	86.31	95.00
		BMI	16.32	1.98	10.52	15.39	16.16	17.60	21.43
[23–29]	112	Height	88.99	4.61	70.00	85.42	87.94	91.63	102.00
		BMI	15.81	2.05	11.23	14.42	15.54	16.97	24.49
[29–35]	111	Height	93.95	4.88	80.00	90.61	93.00	95.85	110.00
		BMI	15.49	2.01	9.99	14.38	15.47	16.79	22.31

### 3.4 Food collection

Of the 1,224 children included, 32 (2.6%) had at least one meal at the day-care center and 68 (5.6%) had at least one meal with the nanny.

The days on which the food diary was completed were distributed as follows: Monday 12.25%, Tuesday 14.7%, Wednesday 13.2%, Thursday 15.0%, Friday 11.5%, Saturday 16.5%, and Sunday 16.9% (*p* < 0.0001). Tuesday and Monday were the most common days for starting the report, with 22 and 19% of entries, respectively, while Sunday (7.8%) and Saturday (9.1%) were the least frequent. As planned, all children had a report that included a weekend day (49% on Saturday and 51% on Sunday), which was more commonly used as the third day. The time between the first and last day of food collection was 4 days for 57% of the children, 5 days for 30%, and less than 7 days for 96% of the children. The number of meals was kept the same each day and ranged from 4 to 7, depending on age. The average number of foods consumed per day was 13.5, with no significant difference between days 2 and 1, days 3 and 2, or days 3 and 1. The food diaries listed 6,008 different foods, including 1,696 specific baby foods, such as infant, follow-on, and young child formula. Up to the age of 4 months, 92% of non-breastfed infants consumed only formula, with an additional 5% consuming drinking water. The reported age for introduction of solid foods (*n* = 889) in the total sample was on average 5.46 ± 2.04 months (median 5.0; interquartile range 4.23–6.0; range 0.23–21.23). After the age of 7 months, nearly all infants (94.65%) consumed solid foods.

## 4 Discussion

### 4.1 Study design and methodology

The Nutri-Bébé 2022 study was designed to assess feeding practices and short-term nutrient intake over three non-consecutive days, providing detailed descriptions of the foods, preparation methods, and quantities consumed by children aged 0.5 to 35 months. The methodology, described in detail above, fully aligns with the latest guidelines of the European Authority (24). Nutri-Bébé 2022 is the sixth survey of this type conducted over the past 41 years using a similar methodology. The key difference in this iteration was that the food diary and final questionnaire were administered online. This change made it easier for investigators to collect data directly and reduced the study’s cost. Additionally, it enabled the implementation of automatic quality controls and the adaptation of tools to assist with accurate data entry, based on feedback (e.g., Q&As). Several studies have evaluated the validity of online dietary assessment methods by comparing them with interviewer-administered recalls and weighed food records (33–35). Online records have been shown to provide similar dietary intake data compared to paper-based versions.

### 4.2 Parental involvement and demographics

The inclusion of fathers was also a new element in the 2022 edition. However, in most cases, the mother considered herself primarily responsible for feeding the child, with fewer fathers than expected (< 3%) responding to the survey. Non-breastfeeding mothers were older than breastfeeding mothers. Overall, mothers tended to be older than in 2013 (31.98 ± 5.45 years vs. 30.8 ± 5.4 years) (23). Household and parent characteristics differed somewhat in 2022 compared to 2013. Among those recruited, the level of education and the socio-professional category of responding parents were higher in 2022. However, this was addressed through weighting in accordance with the characteristics of the general population.

### 4.3 Anthropometric data

Along with the absence of disease required for inclusion, the distributions of anthropometric data indicate good health among the recruited children. The recent French national growth charts have been shown to be close to the WHO charts in the early life period (32, 36). Boys generally had a higher weight than girls, but the difference was significant in only a few age groups, and there was no difference in age distribution. All growth charts indicate that boys are usually slightly heavier than girls at the same age, along with differences in body composition (32, 36, 37). Surprisingly, the prevalence of children who might be undernourished (body weight < 3rd percentile) is higher than expected, while the prevalence of children with a weight > 97th percentile is close to expected. No explanation can be given at this time.

### 4.4 Breastfeeding prevalence and introduction of solid foods

Breastfeeding prevalence was essentially the same as observed in 2013 (23), and lower than that recently reported by three large cohort studies conducted in France. Between 2010 and 2016, the Nutri-Net Santé cohort recruited 34,445 children of all ages, among whom 83% had been breastfed (38). Of the 9,697 infants, including 5.4% preterm newborns, participating in the “Enquête Nationale Périnatale 2021,” 67.1% were breastfed in the maternity ward, and 54.2% of the 6,790 infants followed were still fully or partially breastfed at 2 months of age (39). In the Epifane study 2021, 77% of the 3,534 infants were breastfed in the maternity ward, and 34.4% were breastfed at 6 months (n = 3,323) (40). The age of introduction of solid foods in the Nutri-Bébé 2022 study is comparable to that reported in the 2013 Nutri-Bébé study (5.4 ± 2.1 months) (23). In the Epifane study, the median age of introduction of foods other than milk was younger (4.5 months) (40). In both our study and the Epifane study, the extreme values are surprising and unlikely, suggesting a certain degree of misunderstanding of the question by some parents.

### 4.5 Strengths

The strengths of the current study are as follows: (i) its originality, given the scarcity of studies relating to these age groups and the repetition of the survey every eight years; (ii) the inclusion of a large cross-sectional sample of infants and young children, stratified into 11 age groups to better describe the different stages of feeding practices according to child development; (iii) efforts to ensure the representativeness of the sample as much as possible; and (iv) the collection of food data on three non-consecutive days, including a weekend day, following a face-to-face interview.

The study design contributed to its strength by allowing both validity and reproducibility. It used a quota random sampling method prospectively. Over the years, up to the 2013 edition, the sample size was increased and then remained comparable in 2022, reaching 1,224 participants (23). The robustness of the study was thus improved, and the final sample sizes are high, particularly considering EFSA recommendations (260 infants and 260 young children aged 1–2 years) (24). Compared to other recent surveys, the Nutri-Bébé 2022 survey is also among those involving the largest and/or most detailed (i.e., age groups) samples of children under 3 years old (Table 6) (4, 6, 11, 15, 41–48). Characteristics of older studies have been previously reported (23, 49). The heterogeneity in methodology among these studies has been highlighted.

**Table 6:** Comparison of the Nutri-Bébé 2022 sample with those of other recent studies that included infants and/or young children.

Country	Survey	Years	Reference	Infants	Young children
France	The Nutri-Bébé Survey	2022		707	517
France	The Individual and National Food Consumption survey (INCA 3)	2014–2015	(41)	80	229
Spain	The Nutritional Study in Spanish Pediatric Population (EsNuPI)	2018	(11)	0	374
Netherlands	The National Food Consumption Survey (DNFCS)	2012–2016	(42)	0	232
Serbia	The Food Consumption Survey	2017–2021	(10)	0	290
USA	The National Health and Nutrition Examination Survey (NHANES)	2021–2023	(43)	377, 0–24 months	
USA	The Feeding Infants and Toddlers Study (FITS)	2016	(4)	366	342
18 Latin American countries	The E-1500 survey	2016–2017	(44)	1,284, 0–24 months	
Brazil	The ENANI-2019 survey	2019	(45)	14,558, < 5 years old	
Argentina	The National Survey on Nutrition and Health (ENNyS 2)	2018–2019	(46)	1,622, 6–11 months	2,775, 12–23 months
Australia	The National Nutrition and Physical Activity Survey (NNPAS)	2011–2012	(47)	0	228, 24–35 months
Australia	The Feeding Infants and Toddlers Study (OzFITS)	2020–2021	(6)	126, 0–5 months; 308, 6–11 months	542, 12–23 months
New Zealand	The Eating Assessment in Toddlers (EAT) Study		(48)	0	153, 12–24 months

Regarding representativity, the initial weighting of the results brought them closer to the characteristics of the population from mainland France. The second weighting, performed after oversampling, made the final data representative of those found in the initial weighted sample.

For dietary assessment, the EFSA recommendations of 2 to 3 days of collection were followed, but the time between the first and last day was most often less than 7 days, while EFSA recommends at least 7 days (24). In the EFSA Pilot Study for Assessment of Nutrient Intake and Food Consumption Among Kids in Europe (PANCAKE), a protocol including a 3-consecutive-day food record was used for half of the participants (50). Non-consecutive days are considered more appropriate to estimate the prevalence of inadequate intake (24, 51). Diet records over several days are considered to provide quantitatively accurate information and are regarded as the “gold standard,” with good validity and reliability (16, 17, 24, 52). Repeated food data collection on non-consecutive days, as performed in our study, is recommended to reduce within-person error (17, 49). The use of standardized questionnaires, as in previous studies, helped collect data with more homogeneous quality between participants and provided a precise description of food practices and nutrient intakes.

Thanks to these strengths, the Nutri-Bébé 2022 study will provide a strong database that could be used for other research related to children’s diets. As in the 2005 and 2013 editions, the data will be shared with ANSES to support risk assessment and to update the level of exposure of infants and young children to pesticides and contaminants (53).

### 4.6 Limitations

Despite these strengths, certain limitations in the design and methods of this study can be identified. Like most epidemiological nutrition studies, the Nutri-Bébé studies are observational and subject to various biases, which we attempted to minimize through careful design, consideration of inter-individual variation, a large sample size, and data weighting (54). However, randomized trials of dietary interventions would also have several limitations and are unfeasible for practical or ethical reasons (16, 17). The voluntary nature of sample recruitment and the pre-designated days for food data collection could influence usual feeding practices (17). Additionally, the requirement for parents to have good French-language proficiency and access to the internet to complete online questionnaires has certainly introduced selection bias. As a result, the initial sampling was not fully nationally representative, including more mothers with higher education and likely higher household income. However, the adjustments made through weighting helped to address these issues to some extent. In addition, the voluntary nature of participation and the fact that the data were self-reported might lead to social desirability bias, such as overreporting of healthy food consumption and underreporting of less healthy foods (4). Some of the data were also based on parents’ memory, which may introduce recall bias.

Although data collection occurred after the SARS-COVID pandemic, we were unable to assess its influence on eating habits. During the pandemic, unhealthy eating behaviors were commonly reported (27, 55). On the other hand, increases in breastfeeding, a resurgence of family mealtimes and home-cooked foods, and decreases in some processed foods and sugar-sweetened beverage consumption were described (25, 26, 56). However, to our knowledge, no study has assessed these changes after the pandemic. In addition, recruitment for the study was carried out over three seasons, but more than half of the sampling occurred in the spring. Although seasonal variability in food consumption is well established in adults and in developing countries (16, 57), few variations in children’s diets have been observed in France, apart from a few particularly seasonal foods such as certain fruits or vegetables (41).

### 4.7 Suggestions for future improvement of the method

Based on these considerations, several methodological improvements can be suggested. It would be beneficial for the same team to handle recruitment, data collection, and statistical analysis in collaboration with the involved experts. Recruiting households with a low socio-economic status, particularly among newly immigrated populations, should be prioritized, even if it requires assisting them with data collection. The quality and reliability of the collected data would likely improve if the investigators were trained dietitians. The need for a second face-to-face interview after online data collection should also be considered to ensure greater data reliability. Implementing these improvements would likely increase the study’s cost, which could be a limitation.

## 5 Conclusion

The Nutri-Bébé 2022 study completes a series of similar studies conducted over more than 40 years. The methodology ensures robustness, reliability, and statistical power. It provides detailed demographic, socio-economic, anthropometric, and early feeding data in a large sample of children under 3 years of age. Adherence to EFSA and STROBE-nut standards reinforces its methodological validity and ensures data comparability and reproducibility. Despite acknowledged limitations, it offers a strong representation, improved by data weighting, of the population of children under 3 years of age living in mainland France. However, it does not account for the most disadvantaged population, which should be considered in future studies. Nevertheless, its large sample size and the 3-day food data collection method allow for an up-to-date and accurate analysis of eating practices and nutrient intake in infants and young children. Our findings should help practitioners, researchers, and policymakers in their efforts to improve child nutrition and health. Furthermore, the data collected should allow for the identification of trends in feeding practices and subsequent nutrient intake over the past 40 years. The current study shows that the prevalence of breastfeeding remains low in our country and that recommendations on the age of introduction of solids are generally followed.

## Data Availability

The raw data supporting the conclusions of this article will be made available by the author FP, without undue reservation.
